# Hard Particle Mask Electrochemical Machining of Micro-Textures

**DOI:** 10.3390/ma17204986

**Published:** 2024-10-12

**Authors:** Ge Qin, Haoyu Peng, Yunyan Zhang, Pingmei Ming, Huan Liu, Xiangyang Wu, Wenbang Zhang, Xingshuai Zheng, Shen Niu

**Affiliations:** School of Mechanical and Power Engineering, Henan Polytechnic University, Jiaozuo 454000, China; mkgpeng1520@163.com (H.P.); mingpingmei@163.com (P.M.); liuhuan@hpu.edu.cn (H.L.); 19511988180@163.com (X.W.); 17613821781@163.com (W.Z.); xshuai_zheng@163.com (X.Z.); ns2019@hpu.edu.cn (S.N.)

**Keywords:** electrochemical machining, hard particle mask, conductive particle, insulating particle

## Abstract

The efficient and cost-effective preparation of masks has always been a challenging issue in mask-based electrochemical machining. In this paper, an electrochemical machining process of micro-textures is proposed using hard particle masks such as titanium and zirconia particles. Numerical simulations were conducted to analyze the formation mechanisms of micro-protrusion structures with insulating and conductive hard particle masks, followed by experimental verification of the process. The results indicate that when the hard particles are electrically insulating, metal material preferentially dissolves at the center of the particle gap, and the dissolution then expands over time in depth and towards the particle contact points. Conversely, using the conductive particles as the masks, such as titanium particles, dissolution initially occurs in a ring region centered at the contact point between the hard particle and the anode, with a radius approximately one-quarter of the chosen particle’s diameter (200 μm), and then continues to expand outward.

## 1. Introduction

Surface micro-texture refers to an array of microstructures with specific shapes, sizes, distributions, and arrangements. Micro-textured metal surfaces exhibit a range of desirable properties, including enhanced wear resistance [1,2,3,4], corrosion resistance, superhydrophobicity [5], improved heat transfer characteristics [6,7], anti-fouling, anti-icing, and biocompatibility [8]. The surface texturing technique involves applying micro-textures to the surface of friction pairs in relative motion using suitable processing methods according to the material properties. This enables the modulation of various functional surfaces’ characteristics. Common surface texturing techniques include micro-machining [9,10], laser processing [11], abrasive gas jet machining [12], electrochemical machining [13,14], and so on. Although these techniques can effectively machine surface micro-texture, they often produce many micro-defects, including surface micro-deformation, residual stress, and micro-cracks, which can compromise the surface quality and performance of the workpiece.

Electrochemical machining (ECM) is a technique that employs electrochemical reactions to dissolve metal anodes and process specific shapes on the anode surface through material removal methods [15,16]. Through mask electrochemical machining, an extension of ECM, we utilize a mask to rapidly electrolyze the desired micro-texture array onto the workpiece surface. The superior surface quality achieved with this method has made it one of the most commonly used and promising techniques for surface texture processing. A critical aspect of ECM is the preparation of the mask. Commonly used masks include one-time masks (e.g., photoresist masks, metal passivation masks, colloidal particle masks) and reusable masks (e.g., flexible PDMS masks, interpolar flexible porous filling masks). M. Datta [17,18], Qu et al. [19], and Shin [20] have achieved high-quality micro-texture arrays on workpiece surfaces using the one-time masks, including liquid photoresist, dry film, metal surface passivation film, and colloidal particle masks. However, traditional masks are often time-consuming to prepare, involve cumbersome processes, and high costs, thus limiting the application range of the one-time mask ECM technology.

To address these limitations, Zhu et al. [21,22] proposed active mask ECM technology, which significantly simplifies the process and expands the application range of the mask ECM technology. Subsequently, Chen et al. [23,24], Chen et al. [25,26], Ming [27,28], Qin [29,30,31], etc., developed a series of efficient active mask ECM techniques, including inter-electrode filling, foam cathode, and line cathode masks, further extending the applicability of active mask ECM.

Since Perrin J discovered the ordered arrangement of submicron particles in 1909 [32], the etching and deposition of micro- and nano-structures with colloidal particles, such as alumina and silica, as masks have become research hotspots due to their advantages of having a short processing cycle, low cost, and strong mask adaptability. However, due to small sizes of the colloidal particles, difficulties in forming regular films, and challenges in collecting after use, they are typically employed as disposable masks.

This study proposes a hard particle mask electrochemical processing technique using insulating and conducting particles as the masks. The aim is to develop an electrolytic processing approach with easy mask preparation and repeatability. To understand the role of an insulating and conductive particle mask in ECM, numerical simulations were conducted to analyze the evolution of electric field distribution and microstructure profiles during mask ECM. Additionally, the micro-texture electrochemical machining experiments were performed using electrically insulated zirconia ceramic particles and conductive metal titanium particles as masks. This study discusses the microstructure-forming process and the mechanisms associated with different material particles used as masks, and investigates the fundamental differences and functions of ECM processes with insulating and conductive hard particle masks.

## 2. Method Description and Model Analysis

### 2.1. Method Description

**Figure 1:**
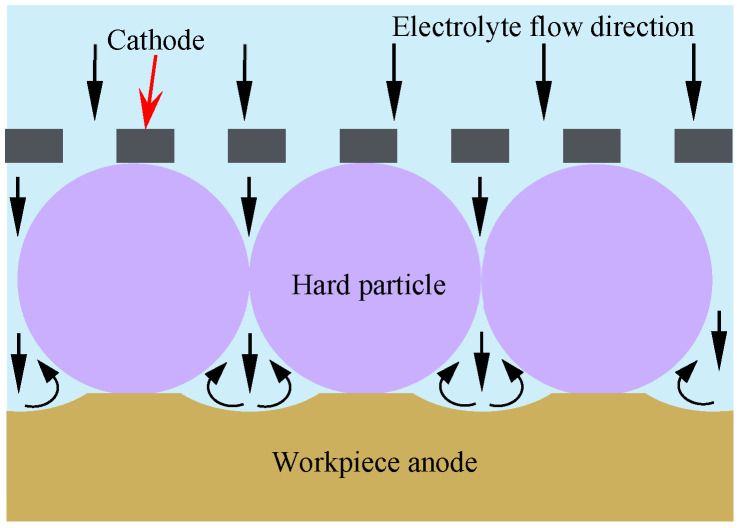
Principle of hard particle mask electrochemical machining.

**Figure 2:**
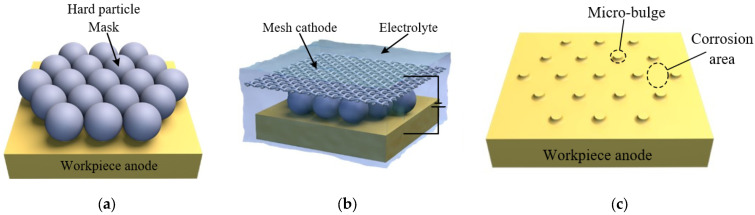
Schematic diagram of hard particle mask electrochemical machining. (**a**) The preparation of hard particle mask, (**b**) ECM process, (**c**) forming of micro-convex structure.

The electrolytic machining is performed using the hard particles as the masks, and a processing principle and schematic diagrams are shown in Figure 1 and Figure 2, respectively. A layer of spherical particles, characterized by their periodic arrangement, was pressed to the surface of the workpiece anode. This was achieved through the clamping effect of a clamping device, which served as a mask for the electrochemical machining process. During machining, the anode material, a corrosion-resistant plate, is selectively dissolved and removed from gaps between the hard particles, the material of which is titanium or zirconia, resulting in the formation of a micro-bulge structure array.

### 2.2. Method Analysis

**Figure 3:**
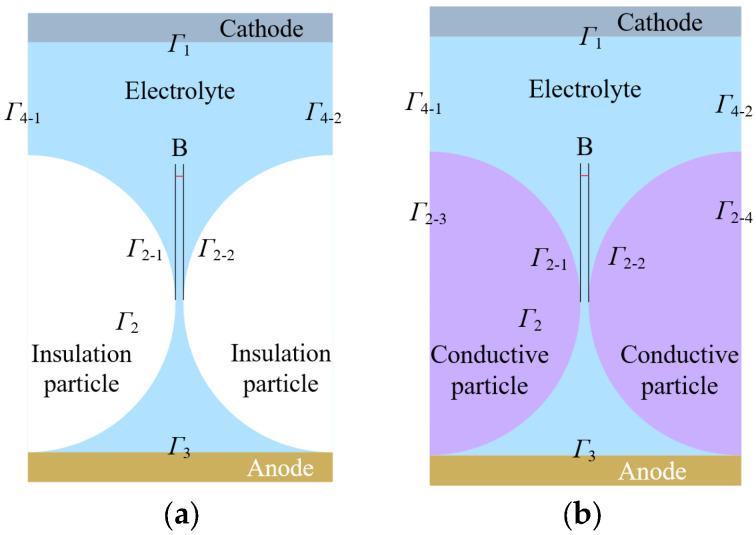
Simulation model of electrochemical machining of hard particle mask. (**a**) Insulation particle simulation model, (**b**) conductive particle simulation model.

To investigate the influence of hard particle mask materials on the mask electrochemical process, this study employs two different types of particles: insulating and conductive particles. In this paper, two-dimensional simulation geometric models of the hard insulating and a conductive particle mask electrochemical machining process are established using COMSOL Multiphysics 6.0 simulation software, as shown in Figure 3. The two hemispheres represent halves of two adjacent particles on the anode surface. The spherical surfaces in the model define the boundaries between the particles and the electrolyte, while the remaining space represents the electrolyte. Figure 3a,b display the simulation models for insulating and conductive particles, respectively, with both particles having a diameter of 200 μm and a processing voltage of 5 V. In Figure 3, Γ_1_ represents the surface profile of the cathode, Γ_2-1_ and Γ_2-2_ represent the surface profiles of the hard particles, and Γ_3_ corresponds to the surface profile of the anode workpiece. Additionally, Γ_4-1_ and Γ_4-2_ denote the boundaries of the closed electric field domain. The boundaries Γ_2-3_ and Γ_2-4_, set for simulation purposes, outline the hard particle but do not participate in electrochemical reactions.

The surfaces of the workpiece anode and the tool cathode are treated as equipotential surfaces. Under these conditions, simulations were conducted to analyze the machining process for both insulating and conductive particles separately.

The Laplace equation for the electric field potential in the electrolyte solution is given as follows:

∂^2^φ/∂x^2^ + ∂^2^φ/∂y^2^ = 0 (1)

φ_a_|Γ_1_ = φ_b_|Γ_2-1_ = φ_c_|Γ_2-2_ = 0 (2)

φ_d_|Γ_3_ = U (3)

∂φ/∂n|Γ_4-1~2_ ≈ 0 (4)

where n represents the normal vector at the boundary, x and y are coordinates of each point in the closed electric field area, and φ is the potential at each point.

When using the conductive particle mask for electrochemical machining, only the boundary conditions change while the geometric model remains the same. Therefore, this paper constructs the same mathematical model for simulation as used in insulating particle processing. The boundary conditions at this stage are described as follows:

φ_a_|Γ_1_ = 0 (5)

φ_d_|Γ_3_ = φ_b_|Γ_2-1_ = φ_c_|Γ_2-2_ = U (6)

∂φ/∂n|Γ_4-1~2_ ≈ 0 (7)

The electric field analysis of the particle mask electrochemical machining is to find the solution of the Laplace Equation (1) satisfying the boundary conditions from Equation (2) to Equations (3) and (4). The electric field analysis of the conductive particle mask electrochemical machining involves solving the Laplace Equation (1) while satisfying the boundary conditions (5)–(7). By doing so, the distribution of electric field intensity distribution can be obtained after the processing.

To analyze the forming process of the microstructure in different materials of hard particle mask electrochemical machining technology, insulating particles/inert conductive particles and conductive particles are selected as masks. Inert conductive particles, composed of titanium metal, form a dense oxide film (the main component of which is titanium oxide) on the surface during the electrochemical machining, and prevent continuous oxidation and corrosion of the titanium. The conductivity of the oxide film is 1 × 10^−7^ S/m. Simulation parameters are listed in Table 1. In order to facilitate the simulation calculation, the following assumptions are made:

1. The electric field between the poles of the external DC power supply is approximated as a steady-state field;
2. The electrolyte concentration remains constant during the processing;
3. The effect of the flow field is not considered.

### 2.3. The Results of Solution

#### 2.3.1. The Influence of Different Conductivity of Hard Particles on the Distribution of Electric Field

**Figure 4:**
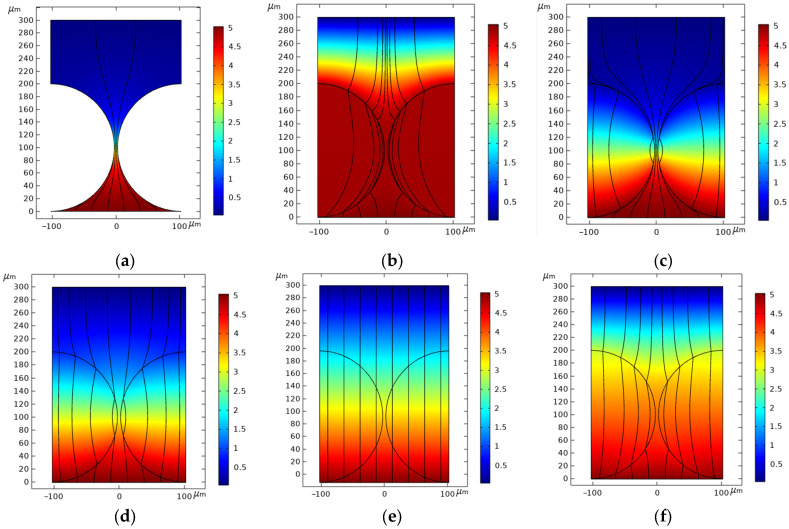
Conductive particles: changes in electrolyte potential and electric field distribution at different particle conductivity κ_1_. (**a**) κ_1_ = 0 S/m(Insulation); (**b**) κ_2_ = 1.7 × 10^5^ S/m, κ_3_ = 1 × 10^−7^ S/m (Titanium and its surface oxide film); (**c**) κ_1_ = 0.1 S/m; (**d**) κ_1_ = 10 S/m; (**e**) κ_1_ = 32 S/m; (**f**) κ_1_ = 100 S/m.

With an insulating particle, no electric field lines pass through the interior of the particle (as shown in Figure 4a). Conversely, when the mask consists of conductive particles, some electric field lines reach the cathode surface through the gaps between the particles, while others pass through the interior of the particles to the cathode surface. As the conductivity of the particles increases at 25 °C, the number of the electric field lines passing through the particles gradually increase (as shown in Figure 4b–f), resulting in a change in the current density distribution on the anode surface. When the resistance value of the particle is lower than that of the electrolyte, which is 20 wt% NaNO_3_ used in our experiments, the electric field lines are primarily concentrated in gaps between the particles, and the maximum current density on the anode workpiece surface occurs between the particles, leading to dissolution predominantly in the spaces between the particles (as shown in Figure 4a,c. For the titanium metal particles used as the mask, a surface film conductivity of 1 × 10^−7^ S/m and an internal conductivity of 1.7 × 10^5^ S/m are used. The electric field distribution follows the trend shown in Figure 4b. According to the principle of electrostatic balance, the charge on the titanium particles in the electric field is distributed only on their outer surface. Consequently, the internal electric field of the titanium particles changes, so only a portion of the electric field lines pass through the particles, while the majority of the electric field lines are concentrated near the outer surface of the particles.

The electrolyte potential between the cathode and the anode varies with changes in the particle conductivity. When the particle is insulating, the electrolyte potential in the gap below the particle radius is approximately 5 V, nearly equal to the anode surface potential, while the potential above the particle radius is nearly 0 V. The electric field lines emitted from the anode surface do not fully return to the cathode, resulting in significant current loss during transmission at the bottom of the particles. Only a small portion of the electric field lines return to the cathode surface due to the minimal resistance provided by the gap between the particles. Consequently, only this portion of the charge contributes to anodic dissolution. As the conductivity of the particles increases, the potential distribution moves upward, causing the potential of the upper half of the particles to gradually increase. Simultaneously, the number of electric field lines passing through the particles gradually increases, and these electric field lines tend to converge toward the particle surface. When the particle conductivity reaches 32 S/m, equal to the electrolyte conductivity, the electric field lines become vertical. A further increase in the particle conductivity results in electric field lines and an electrolyte potential distribution that increasingly resemble those observed with the titanium particles.

#### 2.3.2. Effect of Hard Particle Conductivity on Anode Surface Morphology

**Figure 5:**
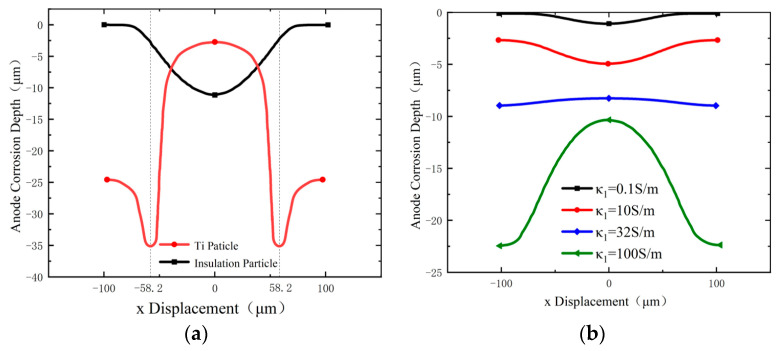
Conductive particles: anode surface morphology changes at different particle conductivity κ_1_. (**a**) Titanium and insulation particle mask, (**b**) different conductivities’ particle masks.

In the simulation, both insulating and conductive particles are used as masks. When titanium metal particles are employed, the particle conductivity is 1.7 × 10^5^ S/m, and the oxide film conductivity is 1 × 10^−7^ S/m, as shown in Figure 5.

With insulating particles, the electric field lines are distributed only within the electrolyte and are concentrated in the gap between the insulating particles. This results in a dissolution morphology on the anode surface that is deeper in the center and shallower on both sides. In contrast, when the titanium metal particles are used, most of the electric field lines are concentrated within the conductive particles due to their significantly high conductivity compared to the electrolyte. Consequently, the electric field lines on the anode surface are concentrated near the outer edge of the contact points between the conductive particles and the anode, rather than in the gaps. This results in a deep groove appearing approximately one-third of the way from of the particle radius in the anode dissolution morphology. Additionally, the current transfer between the two phases is driven by the redox reaction at the interface. The reduction in H^+^ ions at the bottom of the particle gaps generates H_2_, and aggregates OH^−^. The narrow space at the bottom of the particles and the slow electrolyte flow rate hinders the rapid discharge of H_2_ and O_2_, leading to the bubbles on the anode surface, which shields the electric field. As a result, the dissolution in the gap between conductive particles is minimal, consistent with experimental findings.

To explore how varying conductivity affects the anode surface dissolution and groove formation, subsequent simulations were conducted with different conductive particle conductivities. The results indicate that when the conductivity of the conductive particles is lower than that of the electrolyte, the anode surface dissolution morphology is similar to that observed with the insulating particles. As the conductivity of the conductive particles approaches that of the electrolyte, the overall corrosion depth of the anode increases exponentially, and the depression morphology gradually becomes smooth. When the conductivity of the particles equals the electrolyte conductivity (32 S/m), the anode surface dissolution morphology approaches a nearly planar state. When the particle conductivity exceeds the electrolyte conductivity, the anode dissolution morphology begins to approach the simulation results of the titanium particles. The anode corrosion depth at the bottom of the particles exceeds that in the gap between the particles, and grooves begin to form near the contact point between the conductive particles and the anode surface. Therefore, the simulation results suggest that the formation of pits is due to changes in the electric field distribution between the electrodes when the particle conductivity excessed to the solution conductivity.

## 3. Experimental

**Figure 6:**
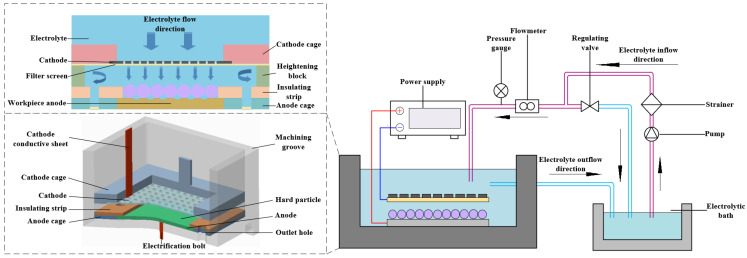
Schematic diagram of hard particle mask electrochemical machining system.

In this paper, an electrolytic processing system using hard particle masks has been developed, as shown in Figure 6. The system includes three main components: a processing unit, an electrolyte circulation unit, and a control unit. The hard particles used in this experiment consist of 95% zirconium oxide and industrial-grade pure titanium particles (Zhufeng-Metar Co., Ltd., Baoji, China) with a spherical diameter of 200 μm. The cathode tool is a stainless steel mesh with a mesh size of 80 and a thickness of 0.2 mm, and the anode is a 304 stainless steel disc with dimensions of φ40 mm × 1 mm and φ20 mm × 1 mm. A 200-mesh nylon filter cloth is employed, and the electrolytic processing tank is machined from acrylic sheet. The processing is conducted in a direct current (DC) mode. Table 2 summarizes the process parameters of these experiments.

The effects of process parameters on the morphology of micro-pit arrays were investigated using the designed device in electrochemical experiments. The surface morphology of the workpiece was analyzed with an ultra-depth field microscope (VHX-2000, KEYENCE, Osaka, Japan) and a scanning electron microscope (SEM, Carl Zeiss NTS GmbH, Oberkochen, Germany).

## 4. Results and Discussion

### 4.1. Surface Morphology of Mask Particle Electrolysis

**Figure 7:**
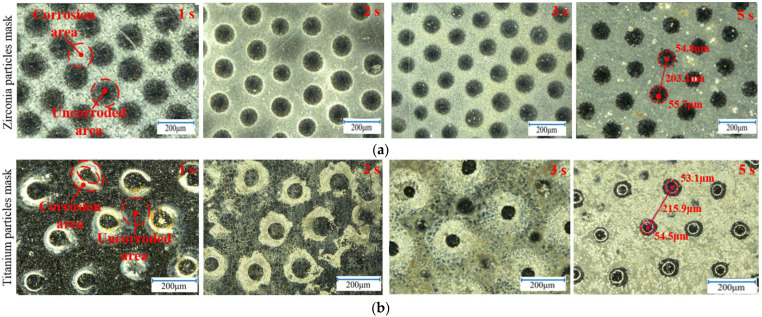
Images of micro-pillar arrays fabricated with different particle mask and electrochemical machining time (T = 1 s, 2 s, 3 s and 5 s, U = 12 V). (**a**) Zirconia particle mask, (**b**) Titanium particle mask.

The electrochemical machining using the hard particle mask was performed with the designed device under the following conditions: an 18 wt% NaCl + 2 wt% NaNO_3_ electrolytic solution, an inter-electrode gap of 0.1 mm, a processing time of 5 s, a temperature of 25 °C and the applied voltage of 12 V. Under these parameters, the micro-protrusion array structures with different characteristics were produced by employing the insulating particles and conductive particles as masking materials for the electrochemical machining (as shown in Figure 7).

With the hard particle mask, electrolytic corrosions proceed selectively on the anode surface, and the morphology of the dissolution zone evolved with an increased electrolysis time. When insulating zirconia particles are used as the mask, dissolution mainly occurs in the unmasked areas between the particles and within particle gaps. Over time, isotropic corrosion expanded the area and depth of the dissolved zone, resulting in a convex plate array structure as the area covered by the particles decreased. In contrast, when titanium metal particles were used as the mask, the dissolution initially occurred around the masked particles, forming ring-like grooves in Figure 7b at 1 s. As electrolysis continued, the dissolution area expands (as shown in Figure 7b at 2 s), until the dissolution zones meet, forming a micro-protrusion array structure.

**Figure 8:**
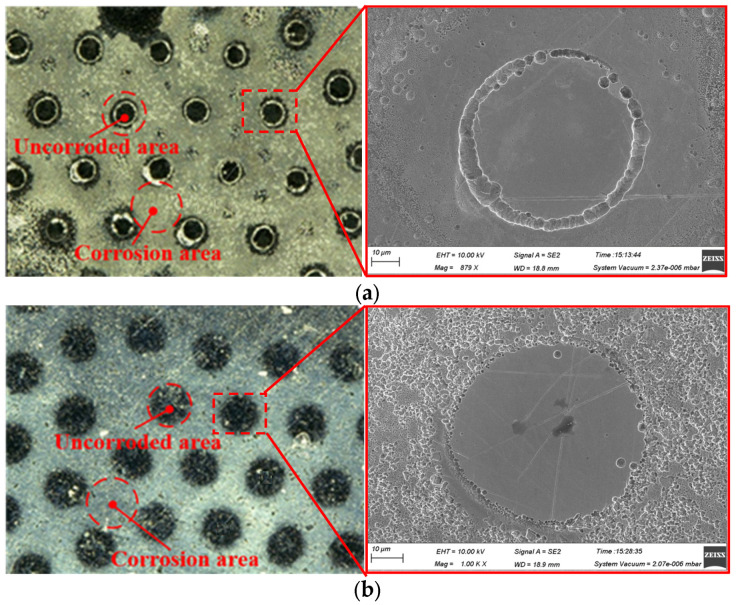
Surface morphology of different surface microstructures electrolytically processed by hard particle mask (D = 200 μm, 10 V, 5 s). (**a**) Titanium particle mask, (**b**) Zirconium oxide particle mask.

Simulation results in Figure 5a show that with the insulating particles, the deepest corrosion occurs between the particles, while with the titanium metal particles, the deepest corrosion occurs around the particles, forming the ring-like grooves. To confirm the reliability of the experimental results, additional experiments were conducted with different experimental parameters, yielding similar outcomes. As demonstrated in Figure 8, electrolytic corrosion at 10 V for 5 s resulted in a ring of groove-like features under the titanium particle mask (as shown in Figure 8a), and a more regular micro-protrusion structure under the zirconia particle mask (as shown in Figure 8b). The uneven bright spots in Figure 8 are caused by dispersion corrosion due to the poor flow of electrolytic products during the electrolysis process. The dimensions of the micro-protrusions were approximately equal to one-fourth of the particle diameters, indicating that the corrosion occurred at one-quarter of the mask particles, consistent with the simulation results shown in Figure 5a.

### 4.2. Micro-Protrusion Forming Principle Using Particle Mask

Through the above simulation analysis and experimental study, it is evident that using the particles with different conductivities as the mask during electrolysis can produce similar micro-protrusion array structures. However, the dissolution mechanisms and processes are significantly different.

**Figure 9:**
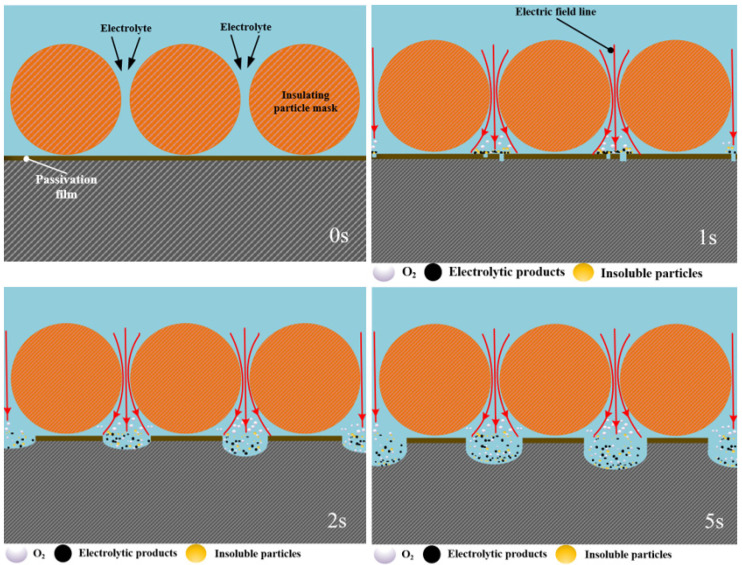
The schematic diagram of the evolution process of the microstructure morphology of the insulating particle mask electrochemical machining.

When the insulating particles are employed as the electrolysis mask, the dissolution initiates in the region between two mask particles. At this location, the passivation film on the anode surface of the workpiece first began to rupture (shown in Figure 9 at 1 s). As electrolysis progresses, extended dissolution occurs, penetrating deeper into the metal and causing the mask area beneath the particle to expand (shown in Figure 9 at 2 s and 5 s), ultimately leading to the formation of the micro-bump array structure. The main reason is that the insulating particles are non-conductive, limiting the flow of current to the gaps between the particles. Consequently, the maximum current density is concentrated at the midpoint between the particles on the anode surface of the workpiece (as shown in Figure 4a and Figure 9 at 0 s), causing the passivation film here to be penetrated at first and subsequent dissolution to start at this location, gradually spreading to the surrounding area.

**Figure 10:**
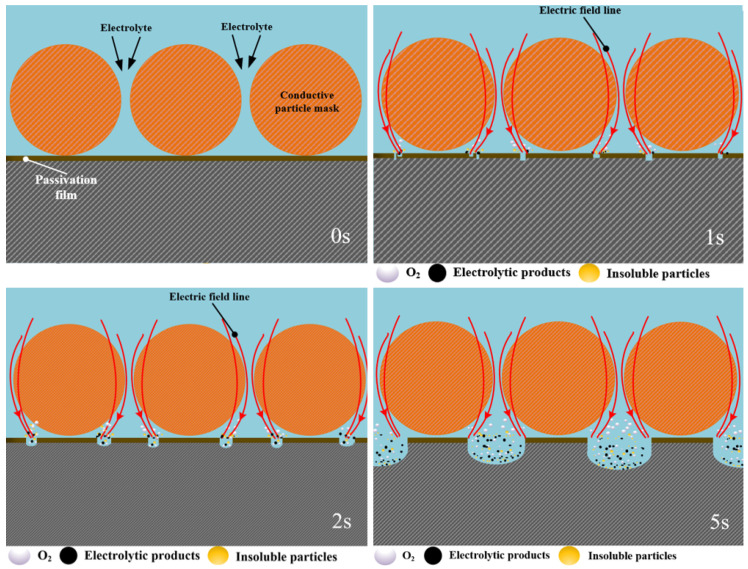
The schematic diagram of the evolution process of the microstructure morphology of the conductive particle mask electrochemical machining.

In contrast, when the metal titanium particles are used as the mask, the surface of the titanium particles undergoes passivation, forming an insulating film in the electrolyte, while the core remains conductive. Electric field analysis indicates that the electric field lines concentrate on the surface of the titanium particles, with some still pass through the interior of the particles. Due to the combined effect of the surface and intermediate current density, the maximum current density on the anode workpiece surface is concentrated at a position covered by the titanium particles rather than being between them (shown in Figure 4b and Figure 10 at 0 s). Consequently, the passivation film here breaks down and is dissolved first, leading to the formation of the ring-like groove structure (shown in Figure 10 at 1 s). Over time, due to the poor mass transfer during electrolysis in the areas where the particles contact the workpiece surface, the dissolution extends towards the depth and the gaps between the particles (shown in Figure 10 at 2 s). When the adjacent grooves diffuse and meet, the micro-protrusion structures are formed (shown in Figure 10 at 5 s).

## 5. Conclusions

This paper presents the electrochemical machining process of micro-textures using hard particle masks, employing titanium particles and zirconia particles as the masks in the electrochemical processing. Under optimized experimental conditions, the titanium metal particles and the zirconia particles were used as the masks to successfully fabricate the micro-protrusion structures with uniform sizes and consistent profiles on the surface of 304 stainless steel. Through the simulation analysis and experiments, the effect of the particle mask’s conductivity on the electrochemical process and the formation of microstructures was investigated, and the fundamental differences of the electrochemical machining using insulating and conductive particle masks was also analyzed, as well as the variations in the microstructure formation process. The experimental results demonstrate that using hard particles as masks for micro-convex processing is feasible. This achievement addresses the limitations of traditional colloidal masks, which are difficult to collect and can only be used once, and exhibits good processing consistency under optimized parameters. It is expected to be applicable to the processing of various workpiece surfaces requiring microstructures. The main conclusions are as follows:

1. Conductive particles, such as titanium, can be used as masks for electrochemical machining.
2. Using the insulating and conductive particles as masks for the electrochemical machining, the formation process of the micro-protrusions differs; however, the micro-protrusion arrays with relatively consistent structural and dimensional characteristics can be achieved. The micro-protrusions obtained from the two particles under the same processing conditions are roughly one-quarter of the particle diameter. When the particle diameter is 200 μm, the micro-protrusion size is about 54 μm.
3. The conductivity of the hard particle mask is the primary factor directly influencing the electric field distribution between the electrodes and the initial anodic dissolution behavior. When the insulating particles are used as masks, the maximum current density on the surface of the anodic workpiece is concentrated between the particles, where the initial dissolution of the anode occurs and uniformly spreads outward, forming the micro-protrusion structure. In contrast, using the conductive particles as the masks, the maximum current density on the anodic workpiece surface is concentrated with approximately a quarter of the particles beneath the conductive particle. The initial dissolution occurs at this point, forming a ring groove around the particle, and then diffuses in both the gap and depth direction between the particles until the micro-protrusion is formed.

## Figures and Tables

**Table 1:** COMSOL simulation basic parameters table.

Simulation Parameters	Value
Particle diameter/D	200 μm
Interpolar spacing/H	300 μm
Voltage/U	5 V
Time/t	1 s
Electrolyte conductivity/κ	32 S/m
Conductivity of conductive particles/κ_1_	0 S/m, 0.1 S/m, 1 S/m, 32 S/m, 100 S/m
Titanium particle conductivity/κ_2_	1.7 × 10^5^ S/m
Conductivity of titanium oxide film/κ_3_	1 × 10^−7^ S/m

**Table 2:** Experimental parameters.

Parameters	Value
Particle diameter	200 μm
Hard particles material	Titanium and zirconium oxide
Cathode	0.2 mm thickness, stainless steel mesh
Anode	φ40 mm and φ20 mm × 1 mm, stainless steel
Electrolyte concentration	18 wt% NaCl + 2 wt% NaNO_3_
Applied voltage	9–12 V
Inter-electrode gap	0.1 mm
Electrolyte flow rate	1 L/min

## Data Availability

The original contributions presented in the study are included in the article, further inquiries can be directed to the corresponding authors.

## Abbreviation

n	normal vector
x	horizontal coordinate
y	vertical coordinate
φ/V	electric potential
U/V	voltage
κ/S·m^−1^	conductivity
